# Th1-Biased Immunomodulation and Therapeutic Potential of *Artemisia annua* in Murine Visceral Leishmaniasis

**DOI:** 10.1371/journal.pntd.0003321

**Published:** 2015-01-08

**Authors:** Mohammad Islamuddin, Garima Chouhan, Abdullah Farooque, Bilikere S. Dwarakanath, Dinkar Sahal, Farhat Afrin

**Affiliations:** 1 Parasite Immunology Laboratory, Department of Biotechnology, Jamia Hamdard (Hamdard University), New Delhi, India; 2 Division of Radiation Biosciences, Institute of Nuclear Medicine and Allied Sciences, Timarpur, Delhi, India; 3 Malaria Group, International Centre for Genetic Engineering and Biotechnology, Aruna Asaf Ali Marg, New Delhi, India; Yale School of Public Health, United States of America

## Abstract

**Background:**

In the absence of vaccines and limitations of currently available chemotherapy, development of safe and efficacious drugs is urgently needed for visceral leishmaniasis (VL) that is fatal, if left untreated. Earlier we reported *in vitro* apoptotic antileishmanial activity of n-hexane fractions of *Artemisia annua* leaves (AAL) and seeds (AAS) against *Leishmania donovani*. In the present study, we investigated the immunostimulatory and therapeutic efficacy of AAL and AAS.

**Methodology/Principal Findings:**

Ten-weeks post infection, BALB/c mice were orally administered AAL and AAS for ten consecutive days. Significant reduction in hepatic (86.67% and 89.12%) and splenic (95.45% and 95.84%) parasite burden with decrease in spleen weight was observed. AAL and AAS treated mice induced the strongest DTH response, as well as three-fold decrease in IgG1 and two-fold increase in IgG2a levels, as compared to infected controls. Cytometric bead array further affirmed the elicitation of Th1 immune response as indicated by increased levels of IFN-γ, and low levels of Th2 cytokines (IL-4 and IL-10) in serum as well as in culture supernatant of lymphocytes from treated mice. Lymphoproliferative response, IFN-γ producing CD4^+^ and CD8^+^ T lymphocytes and nitrite levels were significantly enhanced upon antigen recall *in vitro*. The co-expression of CD80 and CD86 on macrophages was significantly augmented. CD8^+^ T cells exhibited CD62L^low^ and CD44^hi^ phenotype, signifying induction of immunological memory in AAL and AAS treated groups. Serum enzyme markers were in the normal range indicating inertness against nephro- and hepato-toxicity.

**Conclusions/Significance:**

Our results establish the two-prong antileishmanial efficacy of AAL and AAS for cure against *L. donovani* that is dependent on both the direct leishmanicidal action as well as switching-on of Th1-biased protective cell-mediated immunity with generation of memory. AAL and AAS could represent adjunct therapies for the treatment of leishmaniasis, either alone or in combination with other antileishmanial agents.

## Author Summary

Visceral leishmaniasis (VL) is a fatal, vector-borne tropical disease that affects the poorest sections of the society. The currently available drugs are toxic, expensive and have severe side effects. The problem is further compounded by emergence of VL-HIV co-infection and occurence of PKDL after apparent cure. Thus, alternate therapeutic interventions are needed in the absence of vaccines and mounting drug resistance. VL is also characterized by severe depression of cell-mediated immunity that complicates the efficiency of chemotherapeutic drugs. Restoration of the dampened immune system coupled with antileishmanial effect would be a rational approach in the quest for antileishmanial drugs. Plant derived secondary metabolites have been recommended for the containment of antiparasitic disease including leishmaniasis that synergistically aid in lifting the immune suppression. We previously reported *in vitro* antileishmanial activity of n-hexane fractions of *Artemisia annua* leaves (AAL) and seeds (AAS) that was mediated by apoptosis. In this study, we found significant reduction in liver and spleen parasite burden of *Leishmania donovani* infected BALB/c mice upon oral administration of AAL and AAS with concomitant immunostimulation and induction of immunological memory. The immunotherapeutic potentiation by AAL and AAS with no adverse toxic effects validates their use for treatment of this debilitating disease.

## Introduction

Protozoal infections are a worldwide health problem, particularly in the third world countries [1]–[2], and account for approximately 14% of the world's population, who are at risk of infection. Leishmaniasis is considered by the WHO as one of the six major infectious diseases, with a high incidence and ability to produce deformities [3]–[4]. Therefore, finding a safe, effective and affordable treatment for such neglected tropical syndromes is a major concern and of high priority [3]. There are two main forms of leishmaniasis: cutaneous, characterized by skin sores; and visceral, which affects the internal organs (e.g. the spleen, liver, and bone marrow). Visceral leishmaniasis (VL) is the more severe form, causing significant morbidity and mortality, if left untreated. In the current scenario, the disease is associated with the high cost of treatment and poor compliance. In addition, drug resistance, low effectiveness and poor safety have been responsible for retarding the treatment efficacy of current chemotherapy [5]. Concomitant infection with malaria or pneumonia increases the fatality of the illness if not diagnosed and treated in time. The problem of leishmaniasis has been worsened due to parallel infections in AIDS patients [6]–[7]. In the absence of a credible vaccine, there is an urgent need for effective drugs to replace or supplement those in current use. The pentavalent antimony compounds, which constitute the first line of drugs for treatment of leishmaniasis were developed before 1959. The resistance to these drugs is now widespread in Bihar, India where 50–65% patients fail to be treated successfully with normal dose schedule of these first line drugs [8]. The new drugs that have become available in recent years for the treatment of VL are AmBisome, the excellent but highly expensive liposomal formulation of Amphotericin B (AMB) and the oral drug miltefosine, which has now been registered in India. The toxicity of these agents and the persistence of side effects even after modification of the dose level and duration of treatment are, however, severe drawbacks. Drug combinations like miltefosine/paromomycin and SbIII/paromomycin are also ineffectual, as *Leishmania donovani* is known to easily develop resistance [9]. In spite of rapid advances in synthetic chemistry that promises to offer new drugs, natural products continue to play an important role in therapy: Of the 1,184 new drugs registered between 1981 and 2006, 28% were natural products or their derivatives. Another 24% of the new drugs had pharmacophores (*i.e.*, functional groups with pharmacological activity) derived from natural products [10]. Thus, a good starting point to find anti-parasitic natural products would be traditional medicinal plants that have been employed to treat infections, in Asia, Africa or America [11]. For both good scientific reasons and strong pragmatism, the WHO also advocates the use of traditional medicines for the treatment of these tropical diseases [12]. In the quest for new antileishmanial agents with negligible adverse effects, it was thus imperative to focus on alternative systems of medicine [7] including anti-parasitic plant extracts or secondary metabolites derived from them, as an alternative to synthetic drugs.

It is well documented that a defective cell mediated immune response marks the progression of leishmaniasis and restoration of cellular immunity is critical to disease control [13]. The CD4^+^ as well as CD8^+^ T cells have been implicated in resolution of infection [14]. The Th1/Th2 dichotomy of CD4^+^ T cells is also evident in murine VL where the active diseased state is marked by a predominance of Th2 response whereas protection or cure is denoted by a strong Th1 response [15]. Further, recovery from the disease and resistance to reinfection is attributed to generation of long lasting immunological memory, which is dependent upon parasite specific memory T cells [16]. There is substantial evidence signifying that the immune system synergistically promotes the therapeutic efficacy of antiparasitic drugs [17]. Therefore, antileishmanial drugs that can quickly reverse the immune suppression of the infected host and polarize the response towards Th1 phenotype with generation of immunological memory, besides killing the parasites, are desirable.

In the context of our study, many traditional medicinal plants have been shown to possess dual antileishmanial and immunopotentiating activities validating their use in folk medicine [18], [19]. *Artemisia annua* (Asteraceae), a well-known traditional medicinal plant, has been extensively used as antimalarial [20]–[21] and anticancer agent [22]. Recently the *in vitro* and *in vivo* efficacy of artemisinin (one of the constituents of *A. annua, A. indica* and *A. dracunculus*) against hepatocellular carcinoma [23] and experimental VL has been reported [24]. Flavonoids of *A. annua* have been linked to beneficial immunomodulatory activities in subjects affected from parasitic and chronic diseases [25]. The *in vitro* and *ex vivo* leishmanicidal activity of the *A. annua* leaves (AAL) and seed extracts (AAS) has been evaluated previously against *L. donovani* promastigotes and intracellular amastigotes by our group [26]. In the present study, we have explored the immunotherapeutic potential of AAL and AAS against VL in *L. donovani* infected BALB/c mice.

## Methods

### Animal care and housing

Female BALB/c mice aged 6–8 weeks and weighing 20–25 g were used in the present study after prior approval from the Jamia Hamdard Animal Ethics Committee (JHAEC) for the study protocol (Ethical approval judgment number is 459). JHAEC is registered under the Committee for the purpose of supervision and control of experiments on animals (CPCSEA). All animals were individually housed in the Central Animal House of Jamia Hamdard as per internationally accepted norms. The mice were kept in standard size polycarbonate cages under controlled conditions of temperature (23 ± 1°C), humidity (55 ± 10%), 12:12 h of light and dark cycle and fed with standard pellet diet (Ashirwad Industries, Chandigarh, India) and filtered water (*ad libitum*).

### Plant material and extraction

Fresh *A. annua* leaves and dried seeds with floral parts were collected from the Herbal Garden of Jamia Hamdard, washed, air-dried and ground separately and extracted with n-hexane as described previously [26]. The n-hexane extract of leaves (AAL) and seeds (AAS) were concentrated to dryness under reduced pressure at 35°C using a rotary evaporator and the semisolid paste further concentrated in a vacuum dessicator. Dosing solutions were prepared aseptically in dimethyl sulphoxide (DMSO, cell culture grade), and diluted further in PBS (0.02 M phosphate buffered saline, pH 7.2) to achieve a final DMSO concentration not exceeding 0.2%, which is non-toxic. All reagents including AAL and AAS were free of lipopolysaccharide (0.2 ng/ml endotoxin) as determined by the Limulus amoebocyte lysate assay.

### *Leishmania* culture conditions

*Leishmania donovani* (MHOM/IN/AG/83) promastigotes were grown in M199 medium, supplemented with 10% FBS, 2 mM glutamine, 100 units ml^−1^ penicillin, and 100 µg ml^−1^ streptomycin sulfate at 22°C. Late stationary phase promastigotes were obtained after incubation of the parasites for 4–5 days with starting inoculum of 1×10^6^ parasites ml^−1^ [27].

### *In vivo* evaluation of antileishmanial efficacy of *A. annua* extracts in *L. donovani* infected BALB/c mice

Stationary phase *L. donovani* promastigotes were used to infect 6 to 8-weeks old BALB/c mice (2 ×10^7^/animal) through tail vein. Ten weeks post infection, parasite burden was confirmed in three arbitrarily selected animals; after which, mice were randomly assigned into seven groups of 10 mice each (A–F). Group A – Control infected mice without any treatment (INF); Group B - Vehicle control mice that received normal saline orally (VC). Test fractions and compounds were administered to three groups orally: Group C (AAL); D (AAS); and E artemisinin (ART). These groups received three doses (50/100/200 mg/kg body weight {b.w.}) daily for ten consecutive days. Group F - received Amphotericin B (AMB, 5 mg/kg b.w. on alternate days over a 10 day period, intravenously) and served as the positive control. Ten days post treatment, 5 mice per group were euthanized by carbondioxide asphyxiation, liver and spleen parasite burden determined from giemsa-stained multiple impression smears, and expressed as Leishman-Donovan Units (LDU) that was calculated as the number of parasites per 500 nucleated cells x organ weight in mg [28]. Percent reduction of parasite burden was calculated as: (LDU of infected control - LDU of treated mice)/LDU of infected control mice × 100. Cure or protection correlated with a reduction in hepato-splenomegaly and elimination of parasites to negligible levels [28]. Fourteen days-post treatment; the remaining 5 mice per group were sacrificed for evaluating the immunological response.

### Preparation of freeze-thawed and soluble leishmanial antigen

Freeze-thawed leishmanial antigen (FT) was prepared as reported previously [29]. Briefly, stationary-phase promastigotes, harvested after the third or fourth passage in liquid culture, were washed four times in cold 1× PBS and resuspended at a cell density of 2×10^8^ cells ml^−1^. The preparation was frozen and thawed at 80°C (30 min) and 37°C water bath (15 min), alternately for 6 cycles, and stored at −70°C until use. Soluble leishmanial antigen (SLA) was prepared as reported previously [30]. In brief, the freezing-thawing cycles were repeated ten times, and the suspension finally centrifuged (5250 × g, 4 °C, 10 min). The supernatant containing soluble leishmanial antigen (SLA) was harvested and stored at −70°C until use. The protein content in FT and SLA was measured by the method of Lowry *et. al.* [31].

### Determination of DTH

The delayed-type hypersensitivity (DTH) response in control infected and treated mice was determined as an index of cell-mediated immunity. The response was evaluated by measuring the difference in footpad swelling at 24 h, 48 h and 72 h following intradermal inoculation of the test footpad with 50 µl (800 µg ml^−1^) of FT compared to the PBS-injected contra-lateral footpad [14].

### Lymphocyte proliferation assay

Proliferation of splenic and lymphatic lymphocytes as an index of cell mediated immune (CMI) response, was evaluated in spleen and lymph nodes (axilliary, inguinal and popliteal) by trypan blue dye exclusion as well as by carboxyfluorescein succinimidyl ester (CFSE) staining. Spleens from different groups of mice were homogenized and the erythrocytes lysed with lysis buffer (20 mM Tris, pH 7.4 containing 0.14 M NH_4_Cl) at room temperature, 10 min. After centrifugation (1400 × g, 4°C, 10 min), the cells were washed with PBS and resuspended in complete RPMI-1640 medium (supplemented with 25 mM HEPES (pH 7.4), 50 mM 2-mercaptoethanol, 100 U ml^−1^ penicillin, 100 µg ml^−1^ streptomycin and 10% FBS). Alternately, the homogenous suspension of lymph nodes was washed and resuspended in complete RPMI 1640 medium. The viability of both splenic and lymphatic lymphocytes as determined by trypan blue dye exclusion [24] exceeded 95%. For assessment of proliferation, the spleen (5 × 10^6^ cells ml^−1^) or lymph node (2 × 10^6^ cells ml^−1^) cells were cultured at 37°C for 48 h in a humidified atmosphere containing 5% CO_2_ in the presence of 10 µg ml^−1^ SLA or Con A (5µg ml^−1^). Proliferation was ascertained by direct counting of viable cells after trypan blue dye exclusion [32].

Alternatively, for assessment of proliferation by CFSE dilution, the lymphocytes isolated from treated, infected and naïve mice were stimulated with SLA (10 µg ml^−1^) as described above. The lymphocytes (5 ×10^6^ cells ml^−1^) were incubated with 1 µM CFSE. After 48 h, the cells were washed twice with PBS and finally resuspended in PBS. The cells were acquired in a BD LSR II flow cytometer following which the cell population was assessed and contour plots generated after appropriate gating [33].

### Quantification of nitrite

Nitric oxide (NO), a major microbicidal molecule killing intracellular *Leishmania*, is released during conversion of L-arginine into citrulline by nitric oxide synthase that is activated by the Th1 subset of CD4^+^ T cells. The nitrite, the primary, stable and non-volatile product of NO was quantified as an indirect correlate of NO production. The 48 h culture supernatants of peritoneal macrophages of differently treated, infected and naïve mice was analyzed for nitrite contents in the presence or absence of SLA (10 µg ml^−1^) and, in parallel re-stimulated with AAL and AAS (50 µg ml^−1^) as described previously [34]. Briefly, the Griess reagent (1% sulfanilamide and 0.3% *N*-(1-naphthyl) ethylenediamine dihydrochloride in 5% H_3_PO_4_) was added to the culture supernatant at a 1:1 ratio and incubated for 15 min at room temperature. The optical density (OD) was determined at 550 nm using an ELISA reader. Sodium nitrite (NaNO_2_) diluted in culture medium was used to generate a standard curve.

### Determination of IgG isotypes through ELISA

Mice were bled at the time of treatment and at 10 days post treatment, and sera stored at −70°C until use. The specific serum IgG isotype antibody (Ab) response was measured by conventional enzyme-linked immunosorbent assay (ELISA) [14]. Briefly, wells of ELISA plates (Nunc, Roskilde, Denmark) were coated with FT (25 µg ml^−1^) and incubated overnight at 4°C. After washing three times with buffer (20 mM PBS, pH 7.2 containing 0.05% Tween 20), the wells were blocked with 1% BSA for 2–3 h at room temperature. The plate was washed and mice sera at 1,000-fold dilution was added, followed by washing and incubation with isotype-specific goat anti-mouse IgG1 and IgG2a antibody (Sigma Aldrich) at 4°C overnight. The wells were then washed and incubated at 4°C overnight with peroxidase-conjugated rabbit anti-goat IgG (Sigma Aldrich). The wells were washed and incubated with substrate solution (*o*-phenylenediamine dihydrochloride, 0.8 mg ml^−1^ in 0.02 M phosphate-citrate buffer, pH 5.0, containing 0.04% H_2_O_2_) for 30 min, and the absorbance read on an ELISA plate reader at 490 nm.

### Analysis of Th1/Th2 cytokine levels

The Th1 (IFN-γ) and Th2 (IL-4, IL-10) cytokine concentrations in the sera and culture supernatant of lymphocytes from different groups of mice were measured by a bead-based multiplex assay [35]-[36]. This assay used microspheres as the solid support and allowed simultaneous quantification of cytokines in a flow cytometer according to the manufacturer's instructions. Briefly, serum, culture supernatants from SLA-stimulated (10 µg ml^−1^) lymphocytes or the cytokine standards were mixed with equal volume of antibody-coated capture beads and subsequently incubated with biotin-conjugated secondary antibody mixture (anti-mouse) for 2 h at room temperature in the dark. Beads were then washed (400 × g, 4°C, 5 min) and the supernatant was discarded carefully, leaving approximately 100 µl sample in each tube. This was repeated once, and the samples were further incubated with streptavidin–PE for 1 h at room temperature in the dark. After two further centrifugation steps as mentioned above, the beads were resuspended in assay buffer and read on a BD FACS Calibur (BD Biosciences) and analyzed with Cell Quest software. The data were processed using BD CBA software, with results based on a standard concentration curve.

### Lymphocyte phenotyping in spleen

Lymphocyte phenotyping was performed as described previously [37]. The spleens (1/3 of the organ) from differently treated and untreated BALB/c mice were placed in PBS and stored on ice prior to preparation of single cell suspension. The splenic erythrocytes were lysed as described above. After centrifugation (1400 × g, 4°C, 10 min), the cells were washed with FACS buffer (PBS containing 1%FBS). The cell suspensions were refrigerated (4°C) pending staining with antibodies. For each sample, 2 × 10^6^ cells were stained with anti-CD4-FITC and anti-CD8-PE antibodies for 15 min on ice. The cells were then washed and resuspended in PBS for flow cytometric analysis which was performed on a LSR II flow cytometer equipped with DIVA software (Becton Dickinson).

### Intracellular cytokine analysis

Flow cytometry was performed for intracellular analysis of IFN-γ- producing CD4^+^ and CD8^+^ T lymphocytes at the single-cell level. Splenocytes from treated and untreated infected mice were stimulated with 10 µg ml^-1^ SLA for 24 h. Brefeldin A (10µg ml^-1^) was added to the culture and incubated for 1 hr. The cells were washed with FACS buffer and stained with APC and PE conjugated anti-CD4 and anti-CD8 antibody, respectively, washed and fixed with 100 µl of intracellular fixation buffer and permeabilized with permeable solution (BD Pharmingen). The cells were subsequently stained with FITC-conjugated anti-IFN-γ or isotype-matched control monoclonal antibodies, and analyzed on a flow cytometer following acquisition. The CD4^+^ and CD8^+^ T cells were gated individually for determining the population of FITC positive IFN-γ- producing cells [14].

### Phenotypic analysis of co-stimulatory molecules (CD80 and CD86) on antigen presenting cells

Splenic cells from differently treated and untreated BALB/c mice were suspended in RPMI-1640 medium after removing the red blood cells with lysis buffer as described above. Cells (1 × 10^7^ cells ml^−1^) were washed thrice and incubated for 1 h at 37°C on petri plates. After removing the non-adherent T and B cells, the adherent macrophages were collected and washed with FACS buffer. To quantify the expression of co-stimulatory molecules (CD80 and CD86) on CD11b^+^ and F4/80^+^ cells, 2 × 10^6^ macrophages from each sample were stained with PE-labeled anti-CD80, FITC-conjugated anti-CD86 and APC- labeled anti-CD11b or PE-Cy5- labeled anti-F4/80 monoclonal antibodies on ice for 15 min and washed with PBS. Cells were acquired on a BD LSR II flow cytometer equipped with DIVA software (Becton Dickinson) [38].

### Analysis of memory CD8^+^ T cells

Spleen cells were isolated from differently treated and infected BALB/c mice, washed with FACS buffer and incubated for 30 min at 4°C with the following fluorochrome-conjugated anti-mouse antibodies: CD8-APC, CD62L-PE and CD44-FITC (BD Pharmingen), and then fixed with 2% paraformaldehyde. Cell acquisition was performed with a BD LSR II flow cytometer [16].

### *In vivo* toxicity assay

Hepatic and renal functions of BALB/c mice were evaluated in treated and untreated mice as described previously [36]. Fourteen days post-treatment, mice were bled and sera were separated by centrifugation (5000 × g, 4°C, 2-3 min) and stored at -70°C until use. The hepatic and renal functions was assessed by measuring the levels of serum glutamic oxaloacetic transaminase (SGOT), serum glutamic pyruvic transaminase (SGPT), alkaline phosphatase (ALP), urea and creatinine using commercially available kits (Span Diagnostics Ltd.).

### Statistical analysis

All the *in vitro* experiments were performed at least in triplicate. A minimum of five mice per group were used for *in vivo* experiments. The statistical significance of differences between groups was determined as described in the figure legends using ANOVA followed by Tukey's test by graph pad prism 5 software. *P* value of <0.05 was considered statistically significant. Error bars represent the standard error of the mean (SEM). Results are from one of three representative experiments.

## Results

### Antileishmanial activity of AAL, AAS and ART *in vivo*

**Figure 1:**
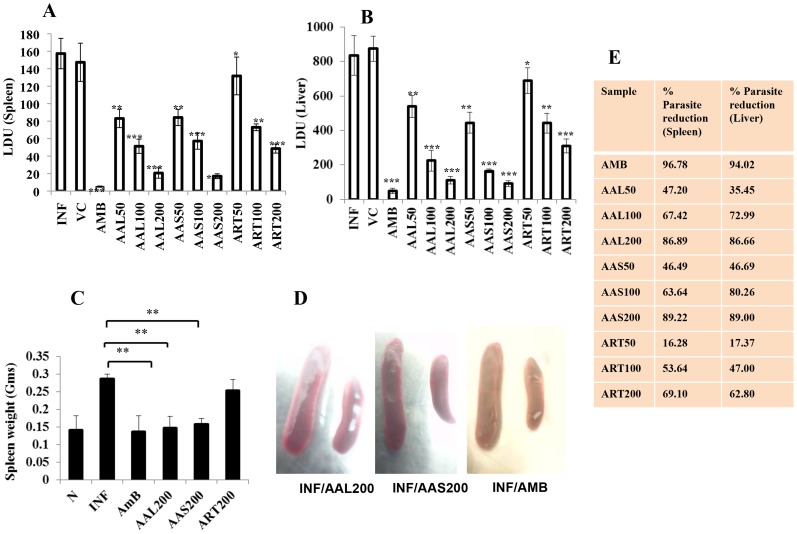
Effect of AAL, AAS and ART treatment on established *L. donovani* infection. Effect of AAL and AAS (50, 100 and 200 mg/kg. bw.) was compared with infection control (INF). 10–week infected BALB/c mice were treated as elaborated in Methods and hepatic (A) and splenic (B) parasite burden were determined at 10 days post treatment by stamp smear method and expressed as LeishmanzDonovan Units (LDU; defined in Materials and Methods). Size (C) and weight (D) of spleen were also determined and compared with infected control (INF). (E) Percent reduction in hepatic and splenic parasite load with respect to infected controls. Untreated and 10-week infected mice were considered as infected controls. Data represent the mean ±SE for five animals per group. Data were tested by ANOVA. Differences between means were assessed for statistical significance by Tukey's test (*, *P* ≤ 0.05; **, *P* ≤ 0.01; ***, *P* ≤ 0.001). Results are from one of three representative experiments.

AAL and AAS administered to 10 weeks infected BALB/c mice at 200 mg/kg b.w. for 10 consecutive days caused 95.45±2.05% and 95.84±1.95% (*P*<0.001) reduction of parasite burden in spleen and 86.67±2.53% and 89.12±1.92% (*P*<0.001) in liver, respectively (**Fig. 1A & B**) at 10 days post treatment. At 100 mg/kg b.w., AAL and AAS induced 88.58±1.23% and 85.08±6.92% protection in spleen and 72.99±7.2% and 80.27±1.25% in liver, respectively. The lowest dose of AAL and AAS (50 mg/kg b.w.) used in this study, caused more than 70% decrease in parasite load in spleen and approximately 50% in liver. ART was comparatively less effective since even the higher dose (200 mg/kg b.w.), could lower the parasite burden in liver as well as spleen by only 50%. With AMB (5 mg/kg b.w.), parasite elimination in liver and spleen was 94.02± 1.81% and 98.09±2.44%, respectively. AAL and AAS treatment (200 mg/kg b.w.) also resulted in significant reduction (48.84% and 45.35% respectively) in spleen weight compared to infected controls (**Fig. 1** **C & D**) that was comparable with AMB (52.33%).

### IgG isotype response in BALB/c mice after cure with AAL, AAS and ART treatment

**Figure 2:**
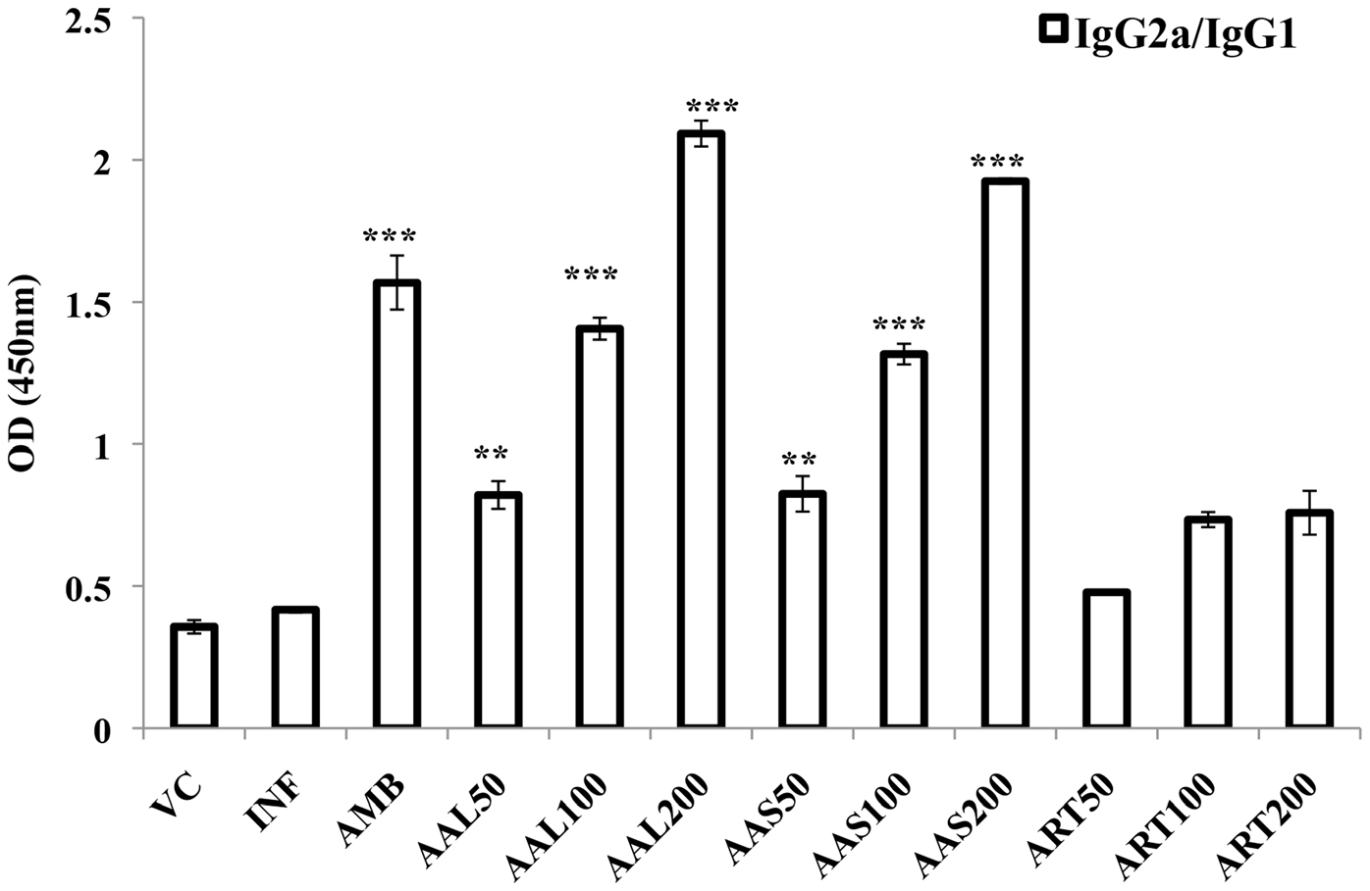
FT-specific antibody response in sera of infected mice upon treatment with AAL (n-hexane fraction of *Artemisia annua* leaves), AAS (n-hexane fraction of *Artemisia annua* seeds), ART (Artemisinin), AMB (Amphotericin B) compared with infected control (INF). Sera from treated and control animals were analyzed for FT specific anti-IgG1 and anti-IgG2a levels by ELISA. Data represent mean ± SE for five animals per group. Data were tested by ANOVA. Differences between means were assessed for statistical significance by Tukey's test (**, *P* ≤ 0.01; ***, *P* ≤ 0.001). Results are from one of three representative experiments.

Since cure of leishmaniasis is associated with an effective immune response, we investigated the possible immunological alterations induced by the treatment of AAL, AAS and ART in *L. donovani*-infected BALB/c mice at cure. Leishmanial antigen (FT)-specific IgG1 and IgG2a isotype levels were assessed in the sera of mice at 10 days post-treatment. Control-infected animals exhibited significantly higher IgG1 than IgG2a levels (*P* ≤ 0.001) compared to treated groups (**Fig. 2**). The highest IgG2a/IgG1 ratio was found in AAL (2.09) and AAS (1.92) -treated mice at 200 mg/kg b.w., followed by AMB treatment group (1.56). In case of mice treated with ART, the IgG1 levels were significantly higher than IgG2a, resulting in decreased IgG2a/IgG1 ratio (0.75) even at the higher dose (200 mg/kg b.w.).

### AAL and AAS in *L. donovani*-infected BALB/c mice induce leishmanial antigen-specific DTH at cure

**Figure 3:**
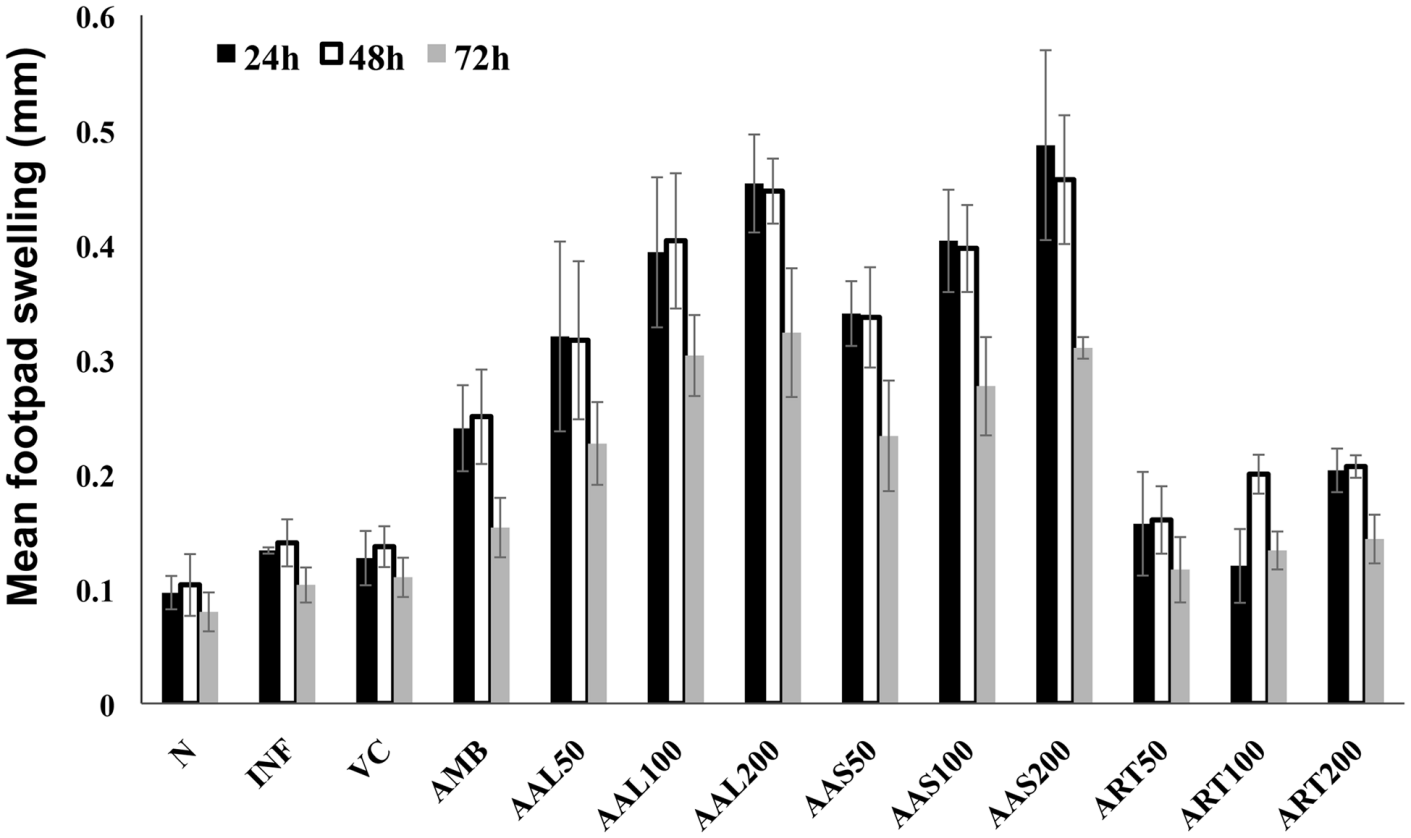
FT-specific DTH responses in infected mice upon treatment with AAL, AAS, ART and AMB compared with the infected control. DTH response was evaluated by measuring the difference between the foot-pad swelling at 24, 48 and 72 h following intradermal inoculation of the test footpad with 50 µl (800 µg/ml) of FT compared with contralateral (PBS-injected) footpad. Data represent the mean ±SE for five animals per group. Data were tested by ANOVA. Differences between means were assessed for statistical significance by Tukey's test (*, *P* ≤ 0.05; **, *P* ≤ 0.01; ***, *P* ≤ 0.001). Results are from one of three representative experiments.

Chemotherapeutic intervention and cure is generally associated with the acquisition of a DTH response and consequently “classical” cell-mediated immunity [39]. Hence, we investigated FT-induced DTH responses in infected BALB/c mice at 10 days post-treatment with AAL, AAS, ART and AMB. AAL and AAS treated mice showed the strongest DTH response at 200 mg/kg. b.w.; a significant increase in footpad thickness was observed at 24 h (0.45±0.07 and 0.48±0.14, respectively) as compared with the INF (013±0.004) control group, followed by 100 and 50 mg/kg b.w. (**Fig. 3**). Whereas AMB (0.24±0.06) and ART (0.20±0.03) treated mice showed a marginal levels of DTH response. There was almost no change in DTH response at 48 versus 24 h; however, the DTH reactivity waned at 72 h in all the groups (**Fig. 3**).

### AAL and AAS treatment induce lymphoproliferation in *L. donovani* infected BALB/c mice

**Figure 4:**
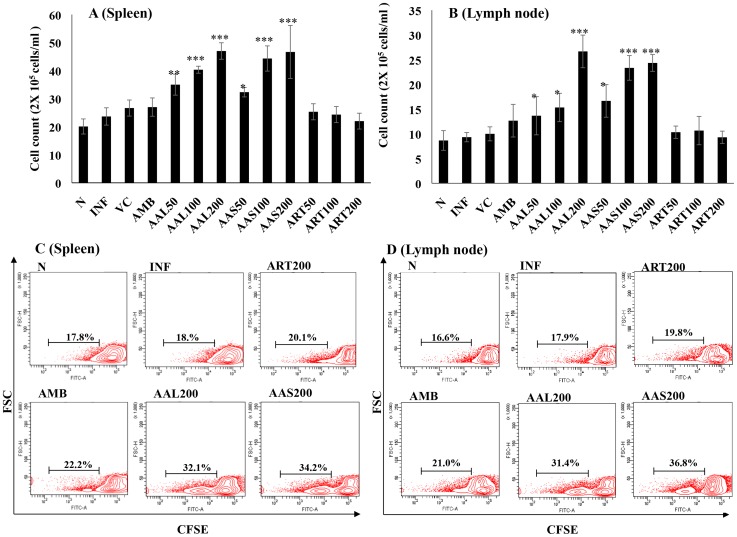
SLA-specific lymphoproliferation in infected mice following cure. 10 days post-treatment, lymphocytes isolated from (A) spleen (5 × 10^6^ cells ml^−1^) and (B) lymph nodes (2 × 10^6^ cells ml^−1^) were plated aseptically and stimulated with SLA (10µg ml^−1^) for 48 h and enumerated microscopically after trypan blue dye exclusion. Lymphocytes isolated from (C) spleen and) (D) lymph nodes of treated, infected and naïve mice were CFSE labeled (in triplicates) and stimulated with SLA for lymphoproliferation by FACS. Data represent the mean ± SE for five animals per group. Data were tested by ANOVA. Differences between means were assessed for statistical significance by Tukey's test (*, *P* ≤ 0.05; ***, *P* ≤ 0.001).

Active VL is characterized by marked T-cell anergy toward leishmanial antigens [40]-[42]. By direct enumeration under microscope, we observed a significant proliferative response of SLA-stimulated splenocytes and lymphocytes from mice at 10 days post-treatment with AAL and AAS. Maximum effect was found at 200 mg/kg b.w. followed by 100 and 50 mg/kg b.w., whereas AMB and ART treatment showed marginal levels of SLA-specific lymphoproliferation in splenic and lymph node cells (**Fig. 4A & 4B**).

Alternately, lymphoproliferative capacity of lymphocytes after treatment with different groups was assessed by CFSE labeling. The percentage of normal cells that underwent division in spleen and lymph nodes was 17.8% and 16.6%, respectively. AAL and AAS treated (200 mg/kg bw) groups exhibited the highest lymphoproliferative response in spleen (32.1% and 34.2%) and lymph nodes (31.4% and 36.8%). AMB and ART treated groups induced low levels of lymphoproliferation (22.2% and 20.1%) in spleen as well as lymph nodes (21.0% and 19.8%) that was slightly higher than that observed in spleen (18.6%) and lymph nodes (17.9%) of infected control group **(** **Fig. 4C & 4D** **)**.

### Nitrite production in cured mice

**Figure 5:**
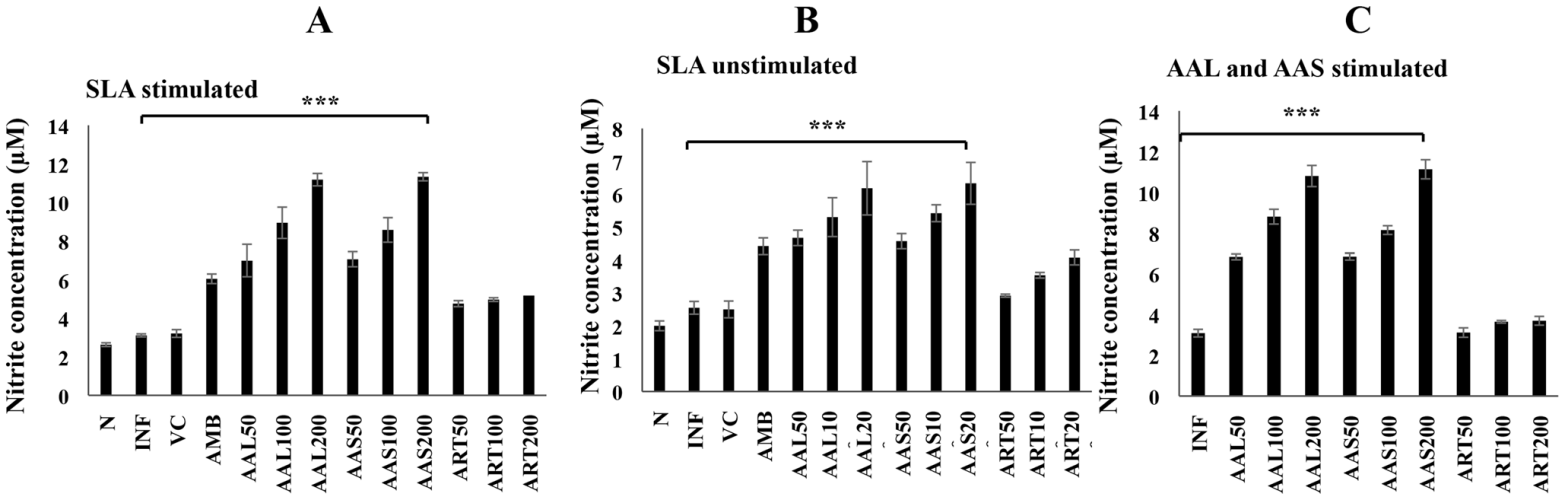
Effect of AAL and AAS treatment on nitrite production in infected BALB/c mice. Macrophages isolated from peritoneal cavity of differently treated, infected and naïve mice and plated (1× 10^6^ cells/well), stimulated with SLA (A) at 10µg ml^−1^, unstimulated (B) and re-stimulated (C) with AAL and AAS for 48 h. Nitrite levels in macrophage culture supernatant of indicated treatment groups were determined by the Griess assay. Data represent the mean ± SE for five animals per group. Data were tested by ANOVA. Differences between means were assessed for statistical significance by Tukey's test (***, *P* ≤ 0.001).

The effect of AAL and AAS on macrophage function was assessed by measuring the amount of Nitric oxide (NO) produced by peritoneal macrophages of treated mice. Griess reagent was used to measure the nitrite levels, the stable end product of NO metabolism. The nitrite concentration (µM) was determined by extrapolation from a standard curve generated with sodium nitrite. In macrophages of AAL and AAS treated mice, a dose dependent NO production was observed upon *in vitro* re-stimulation with SLA followed by AAL and AAS stimulation and un-stimulation. Higher levels of nitrite were produced in AAL (11.18± 0.81µM) and AAS (11.33±0.63µM) treated mice (200 mg/kg/b.w.) after re-stimulation with SLA as compared to INF (3.11±0.09µM) control (**Fig. 5**). In contrast, AMB and ART treatment induced low nitrite levels (6.05±0.20 and 5.18±0.09µM, respectively) in peritoneal macrophages.

### Effect of AAL and AAS on cytokine levels

**Figure 6:**
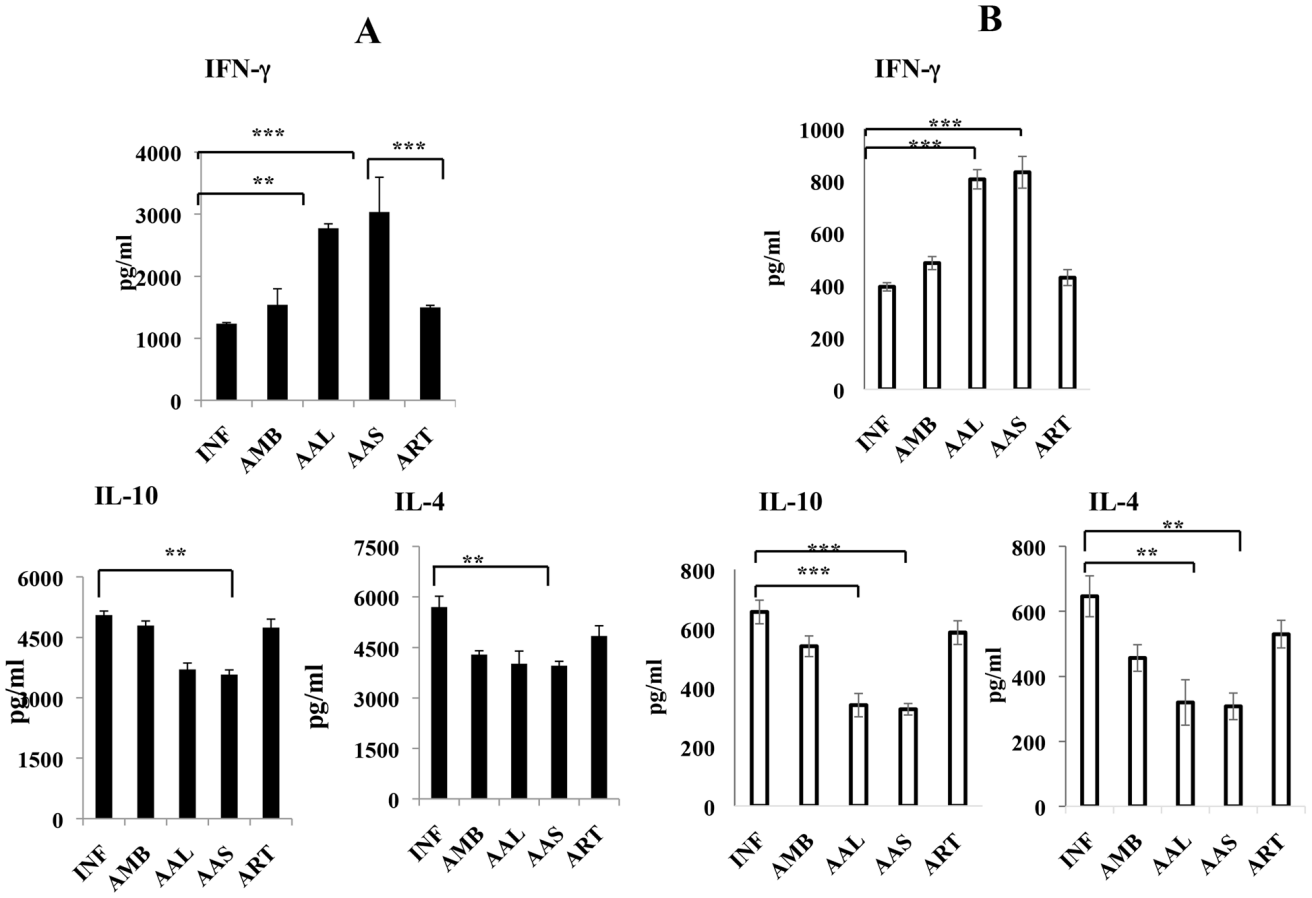
Effect of AAL and AAS on IFN-γ, IL-4 and IL-10 cytokine production. Sera at 10 days post-treatment (A) and culture supernatant (B) from splenocytes of differently treated and infected mice were analyzed for cytokine concentrations by bead-based multiplex assay (ELISA kit, BD Opt EIA set) according to the instructions of the manufacturer as elaborated in Methods. Data are represented as mean ± S.E. of five animals per group. Data were tested by ANOVA. Differences between means were assessed for statistical significance by Tukey's test (*, *P* ≤ 0.05; ***, *P* ≤ 0.001).

To evaluate the immune alterations, Th1 (IFN-γ) and Th2 (IL-4 and IL-10) signature cytokines in serum and culture supernatants were estimated by bead-based multiplex assay. Mice treated with AAL and AAS (200 mg/kg b.w.) induced significantly elevated levels of serum IFN-γ (2771±50.91 and 3033±396.69 pg ml^−1^) and reduced levels of IL-4 (4020±91.92 and 3961.5±208.24 pg ml^−1^) and IL-10 (4231.5±459.27 and 4077.5±35.0 pg ml^−1^) compared to untreated infected controls INF (low IFN-γ; 1236±12.37, high IL-4; 5696±79.9 and high IL-10; 5049±101.47) (**Fig. 6A**). AMB and ART induced low levels of these cytokines compared to INF. Similar pattern of Th1 and Th2 cytokines was observed in the culture supernatant of lymphocytes from mice treated with AAL and AAS and that was significantly high compared with infected (INF) control (**Fig. 6B**).

### AAL and AAS treatment elicited a high population of CD4^+^ and CD8^+^ T cells

**Figure 7:**
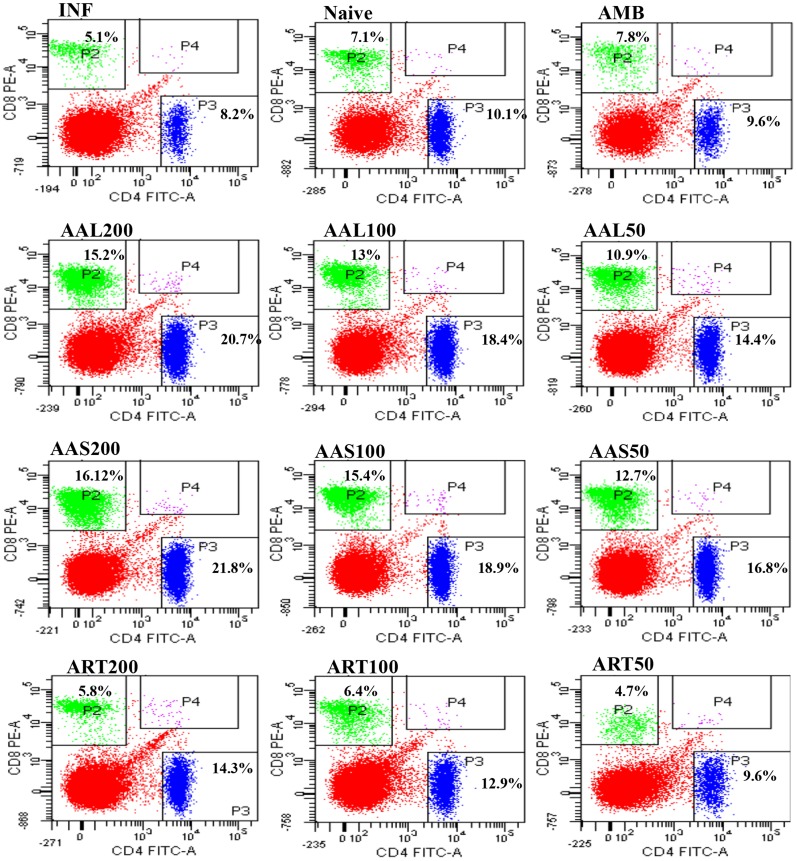
Flow cytometric analysis of CD4^+^ and CD8^+^ T cells of differently treated and untreated infected BALB/c mice. Splenocytes (2 × 10^6^) at 10 days post-treatment were stained with anti-CD4^+^ FITC and anti-CD8^+^ PE antibodies as described in Methods. Data are represented as percent CD4^+^ and CD8^+^ T cell populations.

It is well established that MHC class II-restricted CD4^+^ T cells are dominant during the development of immunity against *Leishmania* [43]. However, a few studies point to an essential role for CD8^+^ cells in immunity to primary infection with *L. major* [44] and also in the induction of long-term, vaccine-induced resistance against many intracellular pathogens [13]. A low population of CD4^+^ (8.2%) and CD8^+^ (5.1%) T cells were detected in the spleens of mice with established *L. donovani* infection (**Fig. 7**). The population of CD4^+^ and CD8^+^ T cells increased 10 days after treatment with 50 mg/kg b.w. of AAL (14.4% and 10.9%) and AAS (16.8% and 12.7%), respectively. The increase was slightly more at a treatment dose of 100 mg/kg b.w. of AAL (18.4% and 13%) and AAS (18.9% and 15.4%). The CD4^+^ and CD8^+^ T cell population was, however, highest at 200 mg/kg b.w. treatment with AAL (20.7% and 15.2%) and AAS (21.8% and 16.12%) (**Fig. 7**). These findings demonstrate a prominent inclination toward Th1 effector function and the involvement of both CD4^+^ and CD8^+^ T cells at cure with AAL and AAS treatment. These responses in case of ART and AMB treated groups were negligible.

### AAL and AAS treatment elicited IFN-γ- secreting CD4 and CD8 T cells

**Figure 8:**
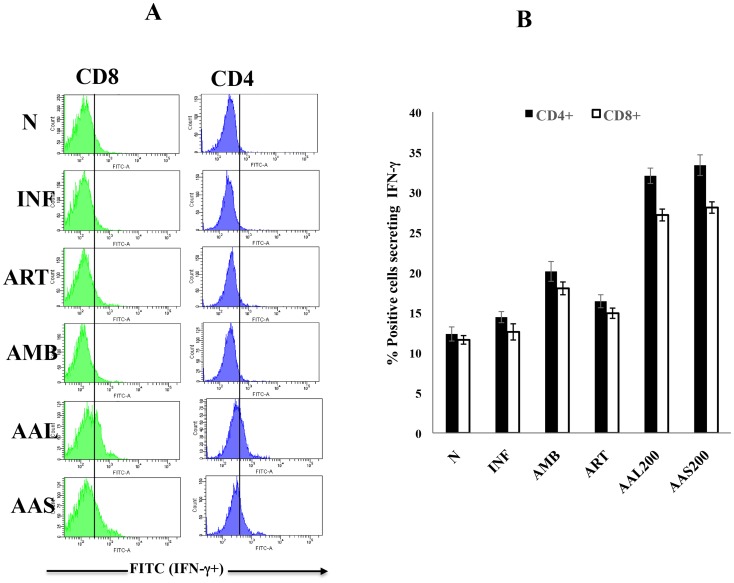
Frequency of IFN-γ producing CD4^+^ and CD8^+^ T cells of differently treated and untreated infected BALB/c mice. Splenocytes were stimulated with SLA (10 µg/ml). Surface phenotyping and intracellular staining were performed as described in Methods, and the cells were examined by flow cytometry. Mean percentages of CD4 and CD8 cells producing IFN-γ in each group of untreated and cured BALB/c mice are presented. The significance of differences between the means was determined by Student's *t* test (***, *P* ≤ 0.001).

Both CD4 and CD8 T cells are source of IFN-γ and are essential for resolution of leishmaniasis [13], [43]. In infected mice, a low frequency of CD4 (14.43±0.40%) and CD8 (12.59±0.58%) T cells secreting IFN-γ was detected which was elevated by AMB treatment (CD4 20.13± 0.71%, CD8 18.02± 0.45%). However, the maximum induction of IFN-γ-producing CD4 and CD8 T cells was observed after AAL (32.05±0.55%, 27.16±0.42%) and AAS (33.37±0.74%, 28.09±0.41%) treatment at 200 mg/kg. b.w. In case of ART (200 mg/kg. b.w) treatment no significant increase in the frequencies of IFN-γ producing CD4 (16.41±0.46%) and CD8 (14.92±0.37%) T cells were observed (**Fig. 8A & 8B**).

### Effect of AAL and AAS on the expression of CD80 and CD86

**Figure 9:**
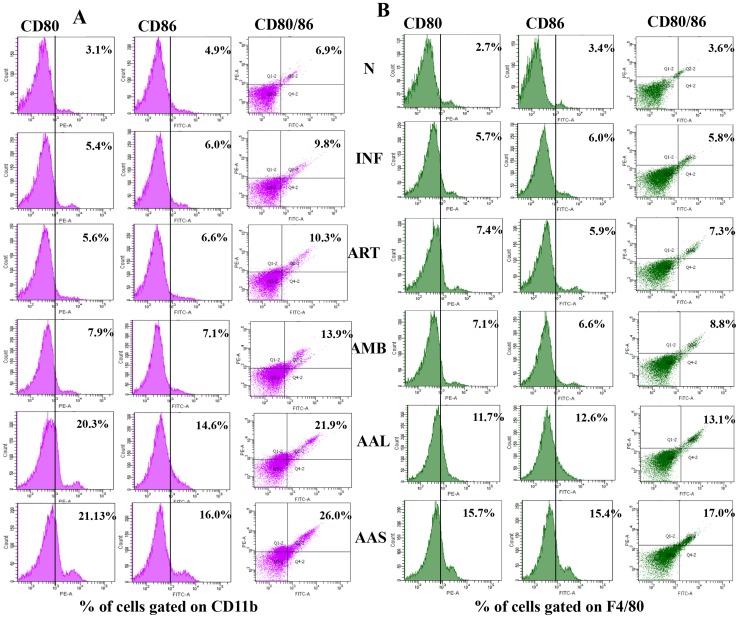
Flow cytometric analysis for the expression of co-stimulatory signaling molecules on splenic macrophages of differently treated and untreated infected BALB/c mice. To quantify the expression and co-expression of CD80 and CD86 on CD11b and F4/80 cells, 2 × 10^6^ macrophages were stained with PE-labeled anti-CD80, FITC-conjugated anti-CD86 and APC-labeled anti-CD11b or PE-Cy5-labeled anti-F4/80 monoclonal antibodies as described in Method section. Cells were gated on F4/80 (A) and on CD11b (B) and the data are represented as percent CD80, CD86 and CD80/86 positive cell populations. Comparison between the infected vs. AAL and AAS treated groups is shown.

Ligands on antigen presenting cells (APCs) called co-stimulatory molecules interact with specific receptors on T cells providing the second signal which then leads to activation of the antigen stimulated T cells. CD80 and CD86 present on APCs are essential for the activation of lymphocytes and the secretion of cytokines. The expression of CD80 and CD86 co-stimulatory molecules was analyzed individually on CD11b (monocytes) and F4/80 (macrophages). CD80 and CD86 expression and co-expression was particularly enhanced on F4/80 gated cells in comparison to CD11b positive cells. AAL and AAS treatment (200 mg/kg.b.w) significantly up-regulated the expression of CD80 (20.3% and 21.13%) and CD86 (14.6% and 16.0%) as well as co-expression of CD80/86 (21.9% and 26.0%) on F4/80 cells. AMB treatment was less effective in up-regulating CD80 (7.9%) and CD86 (7.1%) expression and CD80/86 (13.9%) co-expression, which was followed by ART. ART induced the lowest level of CD80 (5.6%) and CD86 (6.6%) expression and CD80/86 (10.3%) co-expression **(** **Fig. 9A** **)** Similar pattern of CD80 and CD86 expression and CD80/86 co-expression was modulated on CD11b positive cells. AAL and AAS treatment induced maximum up-regulation of CD80 (11.7% and 15.7%) and CD86 (12.6% and 15.4%) expression along with CD80/86 (13.1% and 17.0%) co-expression in comparison with infection control where as in AMB and ART treated groups, the effect was less pronounced **(** **Fig. 9B** **)**.

### Generation of CD44^high^ CD62L^low^ memory CD8 T lymphocytes

**Figure 10:**
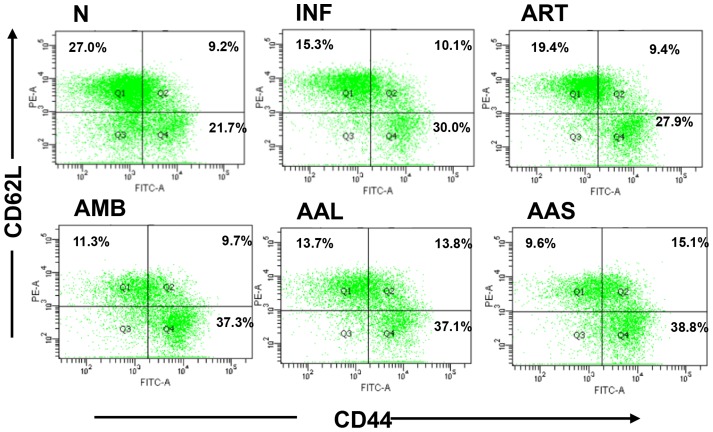
Cytometric analysis of CD62L and CD44 expressing memory subsets on splenic lymphocytes isolated from differently treated and infected mice. 2 × 10^6^ lymphocytes were stained with anti-CD8-APC, anti-CD44-FITC and anti-CD62L-PE. Cells were gated on CD8^+^ T lymphocytes. Comparison between the infected vs. AAL and AAS treated groups is shown.

Resistant to *Leishmania* re-infection is attributed to generation of memory T cells in the host [16]. CD44 CD62L expression was low in infected control group (10.1%), which was up-regulated after treatment with AAL (13.8%) and AAS (15.1%) at 200 mg/kg b.w. demonstrating resolution of infection and generation of memory. AMB (9.7%) and ART (9.4%) treatment exhibited negligible effect on generation of memory T cells. Further the percentage of effector memory cytotoxic T cells (CD44^high^ CD62L^low^) was also increased following treatment with AAL (37.1%), AAS (38.8%) and AMB (37.3%) and no such up-regulation was evident in case of ART (27.9%) treatment **(** **Fig. 10** **).**

### *In vivo* toxicity of AAL, AAS and ART by biochemical estimation of serum enzymes

Estimation of ALP, SGOT and SGPT for liver dysfunction and urea and creatinine for renal dysfunction was done ten days post-administration of AAL, AAS and ART in normal BALB/c mice (**Table 1**) as well as infected and treated mice (**Table 2**). AAL, AAS and ART (up to 200 mg/kg b.w.) treated group demonstrated normal levels of serum enzymes, indicating no *in vivo* toxicity. The fractions thus proved to be non-toxic in BALB/c mice used in antileishmanial screening.

**Table 1:** Effect of AAL, AAS and ART on hepatic and renal functions of normal BALB/c mice.

Group (n = 5)	SGOT (U/L)	SGPT (U/L)	ALP (U/L)	Urea (mg/dl)	Creatinine (mg/dl)
Normal	52.40 ± 6.18	33.09 ± 10.06	86.14 ± 1.59	18.12 ± 3.17	0.96 ± 0.15
AAL 200 mg/kg (oral)	49.29 ± 4.29	30.16 ± 7.60	83.64 ± 3.59	16.69 ± 2.26	0.91 ± 0.09
AAS 200 mg/kg (oral)	47.09 ± 3.26	28.90 ± 5.45	82.19 ± 6.09	17.45 ± 3.64	0.89 ± 0.14
ART 200 mg/kg (oral)	51.46 ± 2.25	32.06 ± 6.18	87.45 ± 3.65	18.10 ± 4.05	1.10 ± 0.11

Mice (n = 5) received AAL, AAS and ART daily for 10 consecutive days. Enzyme estimations (mean± SE) were done using commercial kits.

**Table 2:** Effect of AAL, AAS and ART on hepatic and renal functions of infected and treated BALB/c mice.

Group (n = 5)	SGOT (U/L)	SGPT (U/L)	ALP (U/L)	Urea (mg/dl)	Creatinine (mg/dl)
Infected control	48.40± 5.58	29.78± 3.77	89.42±3.8	15.86± 2.60	1.16± 0.39
AMB 5 mg/kg (i.v)	53.93± 2.78	38.10± 1.16	85.36± 1.7	18.19± 1.90	0.99± 0.14
AAL50 mg/kg(oral)	46.80± 2.99	24.42± 2.56	81.30± 1.9	16.49± 1.00	0.85± 0.10
AAL100 mg/kg(oral)	44.80± 4.28	23.57± 4.13	80.65± 3.9	15.95± 0.98	0.89± 0.09
AAL200 mg/kg (oral)	41.20± 4.94	29.64± 3.36	87.29± 1.2	18.56± 1.61	1.09± 0.13
AAS50 mg/kg (oral)	43.20± 6.52	26.07± 4.90	79.66± 4.2	15.26± 0.80	0.86± 0.08
AAS100 mg/kg (oral)	42.20± 4.23	32.49± 2.27	86.16± 2.6	17.37± 1.20	0.79± 0.08
AAS 200 mg/kg (oral)	43.50± 5.72	29.28± 4.55	82.30± 4.0	16.75± 0.95	1.02± 0.12
ART 50 mg/kg (oral)	44.66± 5.53	29.28± 2.10	84.96± 3.1	17.99± 1.09	0.91± 0.10
ART 100 mg/kg (oral)	43.53± 5.71	28.21± 3.92	83.30± 0.9	17.09± 1.61	0.95± 0.10
ART 200 mg/kg (oral)	44.60± 2.68	32.14± 2.52	88.56± 3.7	18.45± 1.00	1.06± 0.11

Mice (n = 5) received AAL, AAS and ART daily for 10 consecutive days. Enzyme estimations (mean± SE) were done using commercial kits.

## Discussion

In the absence of effective vaccines, emerging resistance against current chemotherapeutic drugs or their combinations, and a stigma of being an AIDS-defining illness, improved therapy for leishmaniasis remains desirable. Plant extracts represent a natural library of potentially bioactive molecules that can activate intrinsic leishmanicidal mechanisms. In our earlier studies, we reported potent anti-leishmanial activity of AAL and AAS with selective elimination of the parasites without affecting host macrophages. The leishmanicidal effect was mediated by programmed cell death. α-amyrinyl acetate, β-amyrine and precursors of artemisinin were the major constituents in AAL and cetin, EINECS 211-126-2 and artemisinin precursors in AAS [26]. In an effort to realize the full therapeutic potential of AAL and AAS, in the present study, we have explored the efficacy of AAL and AAS against VL using *L. donovani* infected BALB/c mice. The major findings emerging from this study are that AAL and AAS (200 mg/kg b.w) result in maximum clearance of parasites (85 to 90%) from the liver and spleen of infected BALB/c mice as compared to untreated infected controls. Significant reduction (88%–96%) in spleen weight was also observed with AAL and AAS. While, only marginal numbers of parasites were cleared from the liver and spleen upon treatment with artemisinin (ART) even at higher dose (200 mg/kg b.w.). Similar therapeutic effect has also been reported with the extracts of *Tinospora sinensis* [45], *Aloe vera* [27], *Actinopyga lecanora* [46] and with the essential oil of *Chenopodium ambrosioides* [47] and *Bixa orellana* [48].

VL is characterized by a variety of immunopathological consequences in man. The most remarkable of these are depression of CMI response and B cell activation [49]. As an index for CMI, DTH, a type IV hypersensitivity reaction was measured in treated mice. DTH develops when antigen activates sensitized T_DTH_ cells resulting in secretion of IFN-γ and IL-2 [50] that promotes enhanced phagocytic activity of the recruited macrophages for effective killing of the parasites. DTH reaction is thus important in host defense system against *Leishmania* parasites. The importance of a positive DTH response in human leishmaniasis is illustrated by the fact that apparent clinical cure in the absence of a positive DTH response is often predictive of a relapsing infection [51]. Our results demonstrated that the DTH response was depressed in *L. donovani* infected BALB/c mice. However, treatment with AAL and AAS stimulated maximum DTH response at 24 h while negligible levels of DTH were induced with ART. Elicitation of DTH response has also been observed with *Asparagus racemosus* extracts [52] and *Prunus cerasus* treatment in infected BALB/c mice.

Resistance against *Leishmania* infection remains largely associated with a polarized Th1 and an insufficient Th2 response. Cytokines such as IFN-γ and IL-4 direct immunoglobulin class switching in B cells to IgG2a and IgG1, respectively [53], as an indirect correlate of T helper subsets potentiated. Thus, IgG2a and IgG1 levels indirectly reflect the Th1/Th2 responses and hence their relative production is used as a surrogate marker for the induction of protective (Th1) or deleterious (Th2) type of immune responses. To assess the immunological status of the mice upon treatment, we evaluated serum levels of parasite-specific IgG1 and IgG2a. *L. donovani* infection in BALB/c mice resulted in increased IgG1 and decreased IgG2a levels. However, treatment with AAL and AAS showed three-fold decrease in IgG1 with approximately two-fold increase in IgG2a levels as compared to infected controls. ART treatment did not reveal any significant difference in isotype levels. Our data reflecting higher levels of IgG2a over IgG1 thus indicate that Th1-mediated protective immunity is generated by AAL and AAS treatment. Our results comply with the reports of Sachdeva *et al.*, [52] who showed decrease in IgG1 coupled with increase in IgG2a levels upon treatment of *L. donovani* infected BALB/c mice with *A. racemosus* in combination with cisplatin. Aqueous extract of *A. racemosus* has also been reported to result in significant increase in antibody titers [15] and upregulation of Th1 and Th2 cytokines [54], suggesting Th1/Th2 adjuvant activity.

Bhattacharjee *et al*., [55] reported that the expression of Th1 signature cytokines (IFN-γ and IL–2) is protective for VL whereas expression of Th2 cytokines *viz.* IL-4 and IL-10 increases during infection. Gomes *et al*. [56] showed that orally administered *Kalanchoe pinnata* selectively suppress IgG and IL-4 and up-regulates IFN-γ production in murine VL. Our studies are in agreement with these observations as AAL and AAS treatment generated a protective immunity through induction of IFN-γ and decline in IL-4 and IL-10 in serum as well as culture supernatants of spleen cells. The percentage of CD4 and CD8 T cells producing IFN-γ also increased after AAL and AAS treatment as depicted by intracellular staining. Further, AAL and AAS (200 mg/kg b.w.) stimulated strong lymphoproliferative responses in lymph nodes as well as spleens, which was observed by CFSE dilution and trypan blue dye exclusion. The increased levels of IFN-γ correlated with the strong proliferative response and activation of Th1 subset of CD4^+^ T cells.

Efficiency of chemotherapy in leishmaniasis is also impaired due to suppression of immune functions during the course of infection [57]. Disease outcome of VL is associated with various immunological dysfunctions. Successful chemotherapy requires strong cellular responses based on CD4^+^ and CD8^+^ T cells. Experimental mouse models of VL show that CD8^+^ T cells are important in control of *L. donovani*/*L. infantum* infection in the liver, through their ability to produce IFN-γ and/or their cytolytic activity [58]. Moreover, CD8^+^ T cells, together with CD4^+^ cells, are required to control and prevent reactivation of VL in mice [59]. *Asparagus racemosus* and *Prunus cerasus* have also been reported to enhance the percentage of CD4^+^ and CD8^+^ T cells in spleen of naive [60] and *L. donovani* infected BALB/c mice upon subsequent treatment [50]. The results of the present investigation revealed that the percentage of CD4^+^ and CD8^+^ T lymphocytes in spleens of *L. donovani* infected BALB/c mice were greatly augmented by AAL and AAS at 200mg/kg b.w. as compared to untreated infected controls as well as ART treated mice. The therapeutic implication of AAL and AAS in VL was further exploited by scoring the memory differentiation markers CD44 and CD62L. AAL and AAS induced generation of immunological memory as characterized by expression of CD62L^low^ and CD44^high^ on CD8^+^ T lymphocytes.

The stimulatory Th1/Th2 balance is dictated by the presence of other costimulatory stimuli simultaneously acting on T cells and antigen-presenting cells (APCs) that play crucial roles in eliminating intracellular pathogens. The optimal activation of naive T cells is achieved by occupancy of T-cell receptor (TCR) by the peptide-MHC complex displayed on the surface of APCs, delivery of co-stimulatory signals, and the presence of pro-inflammatory cytokines [61]. Ligation of CD28 with CD80 and CD86 is known to induce the secretion of IL-6 and IFN-γ by DCs for T and B cell activation, proliferation, and differentiation [62]. CD80 and CD86 expression has been reported to be down modulated in certain diseases [63]-[64]. The expression and co-expression of CD80 and CD86 was analyzed on CD11b^+^ and F4/80^+^ cells. Treatment with AAL and AAS significantly enhanced CD80 and CD86 expression and co-expression on both CD11b^+^ and F4/80^+^ cells however maximum expression was observed in case of F4/80^+^ population. Thus, our results suggest the potential of AAL and AAS in activating the APCs through co-stimulatory signals that eventually help in the generation of effective immune response by secreting various signaling molecules like IFN-γ for subsequent activation, proliferation, and differentiation of lymphocytes. ART treated mice did not show significant expression of CD80 and CD86 co-stimulatory molecules.

Macrophages can be activated by different signals leading to their development into functionally distinct subsets with different disease outcomes. Thus, appropriate activation of macrophages is crucial for eliminating this intracellular pathogen. Macrophage stimulation is mediated by the products of Th1 and NK cells in particular, IFN-γ, which stimulates macrophages to produce inducible nitric oxide synthase (iNOS, also known as NOS2), an enzyme which catalyzes L-arginine to generate NO and citrulline [65]. NO is a toxic molecule that plays a major role in killing intracellular *Leishmania* parasites by the production of reactive oxygen species and generation of peroxinitrite. Such metabolites can cause protein, lipid and nucleic acid oxidation [66]. The function of NO in the leishmanicidal activity of activated macrophages has been demonstrated both *in vitro* and *in vivo* [67]. Our data demonstrate that AAL and AAS treatment in *L. donovani* infected BALB/c mice induces high levels of nitrite in SLA-stimulated macrophages (Fig. 5A) as compared to infection control as well as ART treated mice, suggesting that the inhibitory effect of the AAL and AAS on infection index is mediated by NO. In the absence of SLA, the NO production was muted but after re-stimulation with AAL and AAS, the NO production was upregulated.

The impairment of kidney function and deterioration of liver function by chemotherapeutic agents is recognized as the main side effect and the most important dose limiting factor associated with their clinical use. There is a continuous search for agents, which provide nephro- and hepatic- protection against the renal and liver impairment induced by chemotherapeutic drugs for which allopathy offers no remedial measures. In the current study, AAL, AAS and ART were administered (50, 100 and 200mg/kg b.w.) in normal and *L. donovani* infected BALB/c mice. It was found that serum levels of SGOT, SGPT, ALP, urea and creatinine in treated mice were comparable to those in naive mice, indicating absence of nephro- and hepato-toxicity.

**Figure 11:**
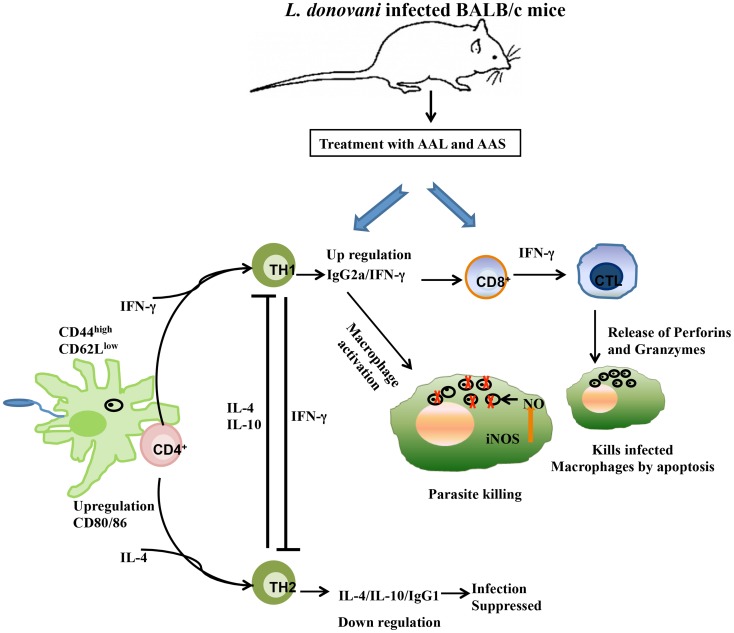
Proposed mechanism for immunomodulatory and therapeutic potential of AAL and AAS.

Taken together, our findings indicate that treatment of infected mice with AAL and AAS significantly decreased the hepatic and splenic parasite load with reduction in spleen weight. AAL and AAS caused increased production of Th1 cytokines (IFN-γ) and concomitant decrease in Th2 signature cytokines (IL-4 and IL-10). The Th1 subset potentiation was also evident from class switching in B cells to produce higher levels of IgG2a over IgG1 and significant elicitation of DTH. AAL and AAS also resulted in higher CD4^+^ and CD8^+^ T cell numbers, lymphoproliferation, up-regulation of co-stimulatory molecules (CD80 and CD86) on APCs and generation of NO. Cure as well as resistance against *L. donovani* infection was due to the parasite killing by AAL and AAS that was mediated by immunopotentiating effects shifting Th1/Th2 balance in favour of the host with induction of cell-mediated immunity as postulated in **Fig. 11**. AAL and AAS may emerge as prospective antileishmanial therapy that may be administered alone or synergistically with current chemotherapeutic drugs, owing to their safety and ability to enhance disease healing Th1 immune responses.
